# Perspectives on brain health and dementia prevention in Latin America: challenges and opportunities

**DOI:** 10.3389/frdem.2023.1275641

**Published:** 2023-10-16

**Authors:** Sarah Fox, Tomás León, Luciano Mariano, Faheem Arshad, Nahuel Magrath Guimet, Grainne Hope, Kuripacha Tituaña, Lina María Zapata-Restrepo

**Affiliations:** ^1^Division of Nursing, Midwifery and Social Work, School of Health Sciences, Faculty of Biology, Medicine and Health, The University of Manchester, National Institute for Health and Care Research (NIHR) Applied Research Collaboration Greater Manchester (ARC-GM), Manchester, United Kingdom; ^2^Global Brain Health Institute, Trinity College Dublin, Dublin, Ireland; ^3^Neurology Department, Memory and Neuropsychiatric Center, Hospital del Salvador and Faculty of Medicine, University of Chile, Santiago, Chile; ^4^Programa de Pós-Graduação em Neurociências, Universidade Federal de Minas Gerais (UFMG), Belo Horizonte, Brazil; ^5^Department of Neurology, National Institute of Mental Health and Neurosciences (NIMHANS), Bengaluru, India; ^6^Global Brain Health Institute, University of California, San Francisco, San Francisco, CA, United States; ^7^Servicio de Neurología Cognitiva, Neuropsicología y Neuropsiquiatría, Neurology Department, Fleni, Buenos Aires, Argentina; ^8^Technical and Technological Training Unit, Pontificia Universidad Católica del Ecuador Ibarra, Quito, Ecuador; ^9^Department of Internal Medicine, Fundación Valle del Lili, Cali, Colombia; ^10^Facultad de Ciencias de la Salud, Universidad Icesi, Cali, Colombia

**Keywords:** brain health, dementia, Alzheimer's, Latin America, opportunities, challenges, perspective, workshop

## Abstract

While age-specific dementia prevalence is falling in many countries, several recent reviews estimate prevalence in Latin America to be higher than anywhere else in the world. This may be, in part, due to the high incidence of socioeconomic and health-related risk factors present in the region. However, growing evidence suggests that primary and secondary prevention via modifiable risk factors is possible, and that 40% of cases may be mitigated through interventions which target modifiable risk. This suggests that there may be significant scope for dementia risk reduction in this region. In June 2021, eight fellows from the Global Brain Health Institute (GBHI) hosted an expert consensus workshop on challenges and opportunities for brain health and dementia prevention in Latin America. The workshop brought together 16 experts in dementia, aging, and brain health from a range of professional backgrounds and geographical regions. From this workshop we collated an expert-led consensus regarding the practical challenges and opportunities implicit in embedding brain health and dementia prevention initiatives in the Latin American context. Here we discuss the outcomes of this workshop, highlighting several challenges and opportunities and discussing how these may be addressed.

## 1. Introduction: local context and goals

We are entering a time where clinical and academic consensus suggests that dementia risk can be modified through management of lifestyle risk factors. This is highlighted in the seminal work of Livingston et al. (2020) which suggests that 12 modifiable risk factors account for 40% of worldwide dementias, an extrapolation of this being that 40% of cases are potentially preventable. Indeed, in several countries age-specific dementia prevalence has fallen, raising hope that this decline may be due to improvements in education, nutrition, health care and lifestyle (Wolters et al., 2020). However, dementia prevalence in Latin America remains stubbornly high. Several recent systematic reviews estimate prevalence in Latin America in the over 60's to be higher than anywhere else in the world (Manes, 2016; Custodio et al., 2017; Parra et al., 2018) and this figure is expected to increase fourfold between 2015 and 2050 placing significant strain on the region's health and economic systems. Concurrent with high dementia prevalence, several socioeconomic and health-related risk factors are also high in Latin America when compared to Canada and the USA. These include a high prevalence of cardiovascular disease, diabetes, an uneven income distribution and lower mean years of schooling (Nitrini et al., 2020). Therefore, there is significant scope for dementia risk reduction in this region.

Here we present the findings of an expert consensus workshop hosted by eight fellows of the Global Brain Health Institute (GBHI) held in June 2021 (all members of the authorship). The online-workshop focused on the challenges and opportunities in developing brain health and dementia prevention strategies across Latin America. Workshop attendees were representative of several professional backgrounds and geographies, this diversity provided a platform for cross-disciplinary discussions and shared learning between several geographical regions and professional backgrounds.

## 2. Methods

In June 2021 a group of 16 experts in dementia and brain health, many living and working in Latin America, attended a 2 h virtual workshop hosted by eight fellows from the GBHI. Attendees were chosen purposefully for their varied professional backgrounds and expertise, which ranged from practicing clinicians, neuroscientists to creative arts and community program practitioners. The aim of the workshop was to discuss the challenges and opportunities present in the Latin American clinical, academic, and societal context which impact the region's ability to adopt brain health and dementia prevention strategies.

The workshop was separated into three sections. In the first, facilitators and attendees were presented with a short introduction and a talk, which was chosen to drive subsequent conversation. This talk was provided by Dr. Lucia Crivelli and broadly covered risk factors for cognitive decline across Latin America, alongside an update on non-pharmacological interventions. Dr. Crivelli is Head of Neuropsychology at Fleni, Buenos Aires, Argentina, her research interests include early detection and neuropsychological biomarkers of Alzheimer's disease, and prevention of cognitive decline.

Attendees were then separated into four break-out groups, each with three or four attendees and two facilitators, groups were specifically selected to ensure members represented a range of professional backgrounds and geographical locations. Group members were given 25 min to discuss and reflect on the following question, set by the eight organizers:

- What social/economic factors (such as education and access to medical expertise) impact on dementia risk in Latin America?

We are particularly interested in your feedback regarding:

1. Your personal and professional experience of the impact these have on people at risk and those living with dementia
2. How this is linked with broader political and structural barriers and
3. how these could be mitigated.

The second section commenced with a talk presented by Carlos Chechetti, a fellow with the Global Brain Health Institute, who spoke about his work as founder and director of Reviving Memories, a social program for older people and Alzheimer's patients. This talk led into our second set of breakout sessions, identical in format to the first and focused upon our second question, set by the eight organizers:

- What are the main challenges/opportunities to implementing brain health/prevention strategies across the region?

We are particularly interested in your feedback regarding:

1. Your personal experience: do you have personal experience, what were the outcomes? Do you believe that your work could be scaled for different populations across the region?
2. What impact do broader political and structural barriers have on the success of such interventions and how could we navigate these?

Finally, all attendees were invited to join as a single large group to reflect on these questions and the discussions they had in their breakout rooms. All discussions were documented during the workshop and later transcribed by GBHI facilitators. Notes from the workshop were collated and analyzed offline, leading to a list of core topics identified by workshop attendees. This list was circulated amongst attendees and facilitators for agreement, refinement, and further comments.

## 3. Workshop feedback and discussion

In this section we discuss feedback from our attendees, this feedback is separated into two core themes:

- Knowledge and Education
- Social and Economic Landscape

Each theme has been further broken down into the challenges and opportunities arising from our discussions at the workshop and also placed in the context of existing knowledge and research into these issues.

### 3.1. Knowledge and education: a challenge

#### 3.1.1. Clinical

Our discussions highlighted that many health and social care workers in Latin America have limited knowledge and training in the field of dementia, especially differential diagnosis (Gleichgerrcht et al., 2011; Arango-Lasprilla et al., 2017; Ibanez et al., 2020, 2021a). In-depth knowledge is often also limited to specialists, such as neurologists and geriatricians. However, our workshop attendees noted that few of these specialists are present in the clinical workforce in most Latin American countries (Gonzalez et al., 2014), an example was given of Brazil where it was suggested that there may be as few as 1 geriatrician per 150,000 patients. The lack of knowledge across different levels of the health and social care system can lead to inconsistent messaging around dementia, therefore making it challenging to harmonize messaging regarding brain health and prevention.

This problem is confounded by a lack of clinical interest in aging and dementia, meaning that few clinicians choose to specialize in gerontology or dementia. This may be underpinned by:

- a preference for working with acute “treatable” conditions which have curative interventions (Prince et al., 2008);
- a focus on the prevention and treatment of communicable diseases such as dengue, tuberculosis, malaria and HIV/AIDS (Gonzalez et al., 2014);
- a pervasive stigma relating to aging and dementia (Ibanez et al., 2020).
- a widespread belief that changes in personality and behavior are the result of natural aging rather than an organic brain disorder (Gonzalez et al., 2014).

Another observation offered by attendees from Brazil and Argentina was that both countries suffered from a lack of trained nursing staff. It was suggested that this deficit was partially due to the cost or nurse training and accentuated by insufficient resources and wealth inequality.

#### 3.1.2. General public

Alongside the assertion that health and social care workers across Latin America have limited knowledge of dementia, workshop attendees also highlighted a lack of understanding amongst the general population.

One attendee observed that, despite available health care provisions and support, many people living with dementia choose not to access these services. This may be linked to the pervasive belief that dementia is a natural part of aging, meaning that those living with dementia and care partners are less likely to seek medical attention or support unless their symptoms are severe (Gonzalez et al., 2014). Another possible explanation may be drawn from findings of a study by Blay et al. which showed that, in a Brazilian cohort, close family was perceived as the most important source of help for someone with Alzheimer's, this being rated above help from healthcare professionals (Blay et al., 2008; Farina et al., 2021). This study also found that a high percentage of survey respondents showed a preference for natural or alternative therapies to treat Alzheimer's. Interestingly, the natural or alternative therapies highlighted in this study included some recommendations now known to reduce dementia risk, including healthy eating, exercising, remaining socially active, and mentally engaged. However, other natural or alternative therapies listed, including the use of vitamins and natural remedies, are less likely to be beneficial and may in some cases have harmful effects (Blay et al., 2008; Livingston et al., 2020). This suggests a lack of consistent public health messaging around aging and dementia and an opportunity for intervention.

### 3.2. Knowledge and education: an opportunity

In our discussions attendees acknowledged that there is a link between the attitudes and experiences of clinical professionals and those of the public. One persistent problem which affects both groups is stigmatization of aging and dementia. Such stigma can lead to a lack of understanding amongst the public and a lack of interest in these fields of study for clinicians. Several attendees suggested that education regarding dementia and brain health should start early and continue at each stage of a student's educational journey. It was suggested that discussing brain health with children early in their education would lead to improved population awareness and knowledge as these children reach adulthood and would also inspire conversations between the children and their families, facilitating a transfer of learning from schools into the family home. Similar projects have been trialed in the UK and have led to positive learning outcomes for both pupils and their families (Watson and Smith, 2020). For such an approach to be successful the geographic and cultural diversity of Latin America must also be considered. It will be necessary to tailor resources to rural and urban populations, involving members of these communities in co-producing materials. For example, advice on exercise and diet must acknowledge the limitations in access to healthcare, produce and recreational activities for some rural communities and tailor brain health advice accordingly; for example suggesting simple “brain friendly” recipes using locally available produce.

It was noted that many people in Latin America are now living longer with a significant proportion of their lives being lived in retirement and “old age.” Therefore, advocacy and empowerment for older people is important. A longitudinal study from Ireland found that older family members are not just receivers of family support and care but also provide essential support to their younger relatives, including essential childcare which allows parents to participate in the labor market (Barrett, 2011). Indeed, similar to Ireland, older adults in Latin America are a source for economic and social support for their families, but unfortunately their contributions are yet to be acknowledged (CELADE, 2021). Given the essential role older adults play in enabling younger individuals to contribute to the workforce, it is important that this role is recognized and celebrated. Therefore, celebrating older people for their contributions to society should be normalized and provisions should be laid out for them to remain physically active and, socially and mentally engaged in their retirement.

### 3.3. Social and economic landscape: a challenge

Socioeconomic inequality and violence are two major concerns in Latin America. Latin America is the most urbanized region of the world, but this urbanization happened in a fast and unplanned fashion, leading to a series of social problems including lack of access to water, basic sanitation, and proper housing. The complexity and history of these sociological factors are beyond the scope of this paper, but these challenges were referenced both directly and indirectly by several of our attendees.

Unfortunately, for many living in Latin America, poverty is a persistent factor impacting many stages of their lives. Beginning in the prenatal period, a substantial portion of Latin American society does not have access to adequate health care, has an unhealthy diet, and limited education (CELADE, 2021). The accumulation of such factors will negatively impact on health in general and in turn brain health, reinforcing a vicious circle of dementia and neurodegenerative conditions. Specifically, in mid-life, inequalities in access to health services also contribute to the development of chronic conditions such as diabetes and cardio-vascular diseases, which are also recognized as risk factors for dementia. This combination of additive risks, starting from a young age, are theorized to contribute to the observation that dementias often arise earlier in Latin America (Nitrini et al., 2009; Xiang et al., 2021).

Another critical issue is the heterogeneity of living conditions and access to resources across the region. Unregulated urbanization brought a series of challenges. A more specialized workforce is often concentrated in the urban areas, which limits access to clinical specialists and diagnostic services for those who live in the rural regions. But, even within cities there are variations in access to and quality of health service. For several reasons, the division of resources across different regions in many cities is not equitable, leading to poor health and social support, which aggravates the burden of social inequality.

### 3.4. Social and economic landscape: an opportunity

Heterogeneity between countries and across regions within the same country is a major challenge for any public policy but also an opportunity to adapt existing policies to different sociocultural contexts. To achieve this, policy makers and researchers should not simply “copy and paste” policies from international protocols but they should involve local populations, from different backgrounds and ethnic groups, in the process of adapting and tailoring protocols. It would be possible then to develop several versions of the same policy, with the same broad aim but tailored to suit a variety of regional and cultural demographics. Such an approach would fit well with the model of brain health diplomacy, through-which threats to brain health are approached and addressed across the life-course by connecting and drawing together learning from experts across a rage of related disciplines, sectors, and regions (Dawson et al., 2020).

Several workshop attendees stressed the importance of developing new tools and measurements to support diagnosis, education, and tailor policy for people living in rural areas, especially those of indigenous descent and individuals with low levels of literacy (Parra et al., 2018). Although this task is not without its challenges, for example developing adapted diagnosis tools and cultural integrated care systems (Parra et al., 2021), progress is already being made in this area. One tool currently in use in other regions is telemedicine (Sekhon et al., 2021), remote meetings and appointments allow non-specialist clinicians and patients to receive guidance and support from specialists based in another part of the country. Assuming provisions can be made to improve regional internet connectivity and to address language and cultural barriers, this may be a cost-effective way to improve specialist clinical knowledge in rural regions. Another way to improve regional dementia knowledge, complimentary to remote/virtual training, would be the creation of online training resources. Variations of a basic training module could be tailored to support clinicians working in different languages and with different ethnic populations. Once the online materials have been created it will be freely accessible and may be used and re-used as necessary, therefore negating many costs involved in face-to-face or virtual training.

To date most policies regarding Brain health are “disease based,” meaning that if you have a particular disease (like Alzheimer's disease) you are entitled to certain treatment. There is need to broaden the focus of these policies, aiming to promote a healthy lifestyle, which can impact on several health conditions (Custodio et al., 2017; Nitrini et al., 2020). The use of taxes as a tool to favor or disfavor certain types of behavior is a commonly used strategy for health promotion. During the workshop, some of the participants recalled the positive experience from taxing tobacco to prevent and stop the pernicious effect this product has over health. Similar schemes can be adopted to stimulate health in general, and, naturally, brain health (Mytton et al., 2007). Tax regulation could be used to stimulate healthier choices, changing eating habits and incidentally mitigating health issues. In fact, some countries/regions in Latin America are using taxation as an alternative to deal with current concerns in the consumption of sugar (Carriedo et al., 2021) and fatty acids (Perez-Escamilla et al., 2017). Such policies could impact on several disease areas, from diabetes to dementia. Therefore, the links between healthy lifestyle choices and a broad reduction in population disease burden should be made explicit to policy makers, including estimations of the economic impact of implementing these policies.

## 4. Conclusions

### 4.1. Steps toward implementation

Findings laid out in the Lancet commission's publication in 2021 (Livingston et al., 2020) and subsequently recognized globally via several influential publications including The World Alzheimer's Report and the WHO's Global action plan on the public health response to dementia (WHO, 2017; ADI, 2021), strongly suggest that primary and secondary prevention via modifiable risk factors is possible and should be recommended as part of clinical best practice. Our workshop identified a range of regional-specific and broader opportunities to raise brain health awareness, encourage brain-healthy activities/lifestyles and to facilitate early differential diagnosis. Numerous practical suggestions as to how this could be achieved were posited by the group, including:

- Reduced tax on healthy foods
- Provisions to encourage older people to remain active in their retirement.
- Improved education for both the general population and practicing experts (clinicians and care workers)
- Targeted education and training for high-risk groups and the professionals who work with these individuals.
- Creative interventions to be made accessible to day-care and support centers.
- Development of diagnosis and support innovations in telemedicine alongside policies to improve internet access for those in rural regions.

However, one clear message supported by most attendees was: to make a tangible difference across the myriad of clinical, social, and political systems involved in this area, strong cross-disciplinary collaborative efforts would be required. Attendees reflected that this workshop had been beneficial in creating a platform for cross-disciplinary discussions but that further opportunities should be created to continue and broaden these conversations. A strong argument was also put forward for including members of the public and people living with dementia in future collaborations and workshops. The power of personal narratives and storytelling to influence political decision making was recognized as being central to any effort to win the “hearts and minds” of local and regional policy makers (Davidson, 2017).

Overall, our discussions suggest the utility of a health diplomacy approach to embedding brain health into the Latin American health and dementia narrative (Dawson et al., 2020). Therefore, necessitating collaborative efforts to overcome systemic challenges and address unmet needs. Such an approach is timely, given the current global focus on brain health and prevention (CELADE, 2021; Xiang et al., 2021), the development and ongoing success of Multi-Partner Consortia and networking initiatives such as ReDLat (Ibanez et al., 2021b), GBHI and emerging evidence for the benefits of arts and creativity for dementia (Gallacher, 2021), and public involvement in dementia research (Pickett and Murray, 2018).

### 4.2. Next steps

Our workshop has taken a first step in initiating a timely and important conversation regarding challenges and opportunities for brain health and dementia prevention in Latin America. However, further work is necessary to recognize the potential in these discussions. We suggest that the following steps will be necessary to translate the opportunities discussed here into practice.

- Development of a Latin American Brain Health Network. This network should incorporate expertise from a range of academic and social fields, including clinicians, researchers, social care workers, creative practitioners, and members of the public living with dementia. This network should also work closely with existing research and brain health networks including GBHI and ReDLat.
- Prioritize and distill several achievable and measurable aims. These may be drawn from the themes emergent from this workshop and finessed through broader consultation.

## Data availability statement

The raw data supporting the conclusions of this article will be made available by the authors, without undue reservation.

## Author contributions

SF: Conceptualization, Data curation, Formal analysis, Funding acquisition, Investigation, Methodology, Project administration, Supervision, Writing—original draft, Writing—review and editing. TL: Conceptualization, Data curation, Formal analysis, Writing—original draft, Writing—review and editing. LM: Conceptualization, Data curation, Formal analysis, Writing—original draft, Writing—review and editing. FA: Conceptualization, Data curation, Formal analysis, Writing—review and editing. NM: Conceptualization, Data curation, Formal analysis, Writing—review and editing. GH: Conceptualization, Data curation, Formal analysis, Writing—review and editing. KT: Conceptualization, Data curation, Formal analysis, Writing—review and editing. LZ-R: Conceptualization, Data curation, Formal analysis, Writing—review and editing.
